# The Effects of 6-Month Aqua Aerobics Training on Cardiometabolic Parameters in Perimenopausal Women—A Randomized Controlled Trial

**DOI:** 10.3390/biology12040588

**Published:** 2023-04-12

**Authors:** Katarzyna Sobczak, Krystian Wochna, Katarzyna Antosiak-Cyrak, Katarzyna Domaszewska

**Affiliations:** 1 Department of Swimming and Water Lifesaving, Faculty of Sport Sciences, Poznan University of Physical Education, Królowej Jadwigi Street 27/39, 61-871 Poznań, Poland; 2 Department of Physiology and Biochemistry, Faculty of Health Sciences, Poznan University of Physical Education, Królowej Jadwigi Street 27/39, 61-871 Poznań, Poland

**Keywords:** red blood cell distribution, visceral adiposity index, aqua aerobic

## Abstract

**Simple Summary:**

Perimenopause begins on average four years before a woman’s last menses. At the end of this time, oestrogen levels drop, which leads to physiological and anatomical changes which have a significant impact on the quality of life. Increase in the risk of coronary heart disease (CHD) during the sixth decade of life can be explained by not only oestrogen deprivation, but also an effect on the lipid profile. A study showed that in healthy women, total cholesterol levels increase on average by 25 mg/dL (14%) and the levels of LDL cholesterol increase on average by 20 mg/dL (19%) from 4 years before to 1 year after menopause, which results in a greater atherogenic profile in postmenopausal women. Heart failure (HF) and cardiovascular disease, the risk of which increases during perimenopause, are not considered isolated conditions, but rather complex systemic disorders. Engaging in the amount of moderate physical activity recommended by the World Health Organization (WHO) helps reduce the risk of death and adverse health events. Regular physical activity reduces body weight, improves cardiopulmonary fitness, and reduces inflammation. The aim of the present study was to assess the effect of a 6-month aqua aerobics programme on cardiometabolic (anthropometric and biochemical) parameters in perimenopausal women.

**Abstract:**

Background: Menopause is a time when women experience a number of physiological and anatomical changes resulting from a decline in ovarian function. It can be concluded that cardiovascular disease increases in perimenopausal and postmenopausal women, irrespective of age-related changes. Engaging in the amount of moderate physical activity recommended by the World Health Organization helps reduce the risk of death and adverse health events. The aim of the present study was to assess the effect of a 6-month aqua aerobics programme on cardiometabolic (anthropometric and biochemical) parameters in perimenopausal women. Methods: In this study, 30 women (control group—16, study group—14) participated in the 6-month aqua aerobics training programme. The mean age of women was 47.67 ± 6.79 year and BMI 26.33 ± 3.64 kg/m^2^. At the beginning and at the end of the study, anthropometric and blood samples analysis were performed. In the blood, lipid profile, morphotic elements were determined. Body composition, waist–hip ratio (WHR), visceral adiposity index (VAI), blood pressure (BP) were measured. Results: The aqua aerobics programme resulted in a significant decrease in the WHR (*p* < 0.05; ES: 2.143), diastolic blood pressure (DBP) (*p* < 0.05; ES: 1.005), and platelet-to-lymphocyte ratio (PRL) (*p* < 0.05; ES: 0.460) and an increase in haemoglobin (HGB) concentration (*p* < 0.05; ES: 0.643). Conclusions: The type of physical activity described in the present study is a great way for perimenopausal women to take care of their overall well-being. The reduction in selected cardiometabolic parameters is important from the point of view of the protection of women’s health.

## 1. Introduction

Menopause is a time when women experience a number of physiological and anatomical changes resulting from a decline in ovarian function. It can be a challenging time for women due to the troublesome physical and mental symptoms that menopause can bring, such as vasomotor symptoms, difficulty focusing and concentrating, skeletal and urogenital problems and burning mouth syndrome, which have a significant impact on the quality of life [1,2]. Moreover, menopause can bring an increased risk of cardiovascular disease, diabetes and breast cancer [3]. Most women experience certain characteristic symptoms both during and before menopause. Perimenopause begins on average four years before a woman’s last menses. [4,5]. In some women, perimenopause can last several months. At the end of this period, oestrogen levels drop, which leads to a wide range of symptoms [4]. Several sex steroid hormones, the activity of the insulin-like growth factor (IGF) system and leptin blood concentration may be closely linked and may modulate cardiovascular risk [6]. The protein that is bound to most circulating IGF (>90% in adult serum) is insulin-like growth factor binding protein 3 (IGFBP-3). It has been shown in studies that elevated IGFBP-3 levels may play an important role in the development of carotid atherosclerosis in the hypertensive patient. High IGFBP-3 levels were associated with a ninefold higher risk of atherosclerotic plaque formation in the carotid arteries compared with LDL cholesterol or IGF-1 levels [7].

One study including women who participated in the Third French cross-sectional MONICA survey on cardiovascular risk showed that the increase in the risk of coronary heart disease (CHD) during the sixth decade of life can be explained by not only oestrogen deprivation, but also an effect on the lipid profile [8]. A study by Fukami et al. showed that in healthy women, total cholesterol levels increase on average by 25 mg/dL (14%) and the levels of LDL cholesterol increase on average by 20 mg/dL (19%) from 4 years before to 1 year after menopause, which results in a more atherogenic profile in postmenopausal women [9]. Oestrogen is involved in the relaxation of blood vessels, helping to accommodate blood flow. Thus, reduced oestrogen levels result in stiffer blood vessels. The ageing of ovaries also results in the activation of the renin–angiotensin–aldosterone system, which leads to endothelial damage, increased inflammation and immune dysfunction. These changes are associated with obesity, diabetes and hypertension [10]. Inflammation involves increased expression of pro-inflammatory cytokines, such as interleukin 6 (IL-6), tumour necrosis factor alpha (TNF-α) and C-reactive protein (CRP). Other, less commonly used inflammatory markers are white blood cell ratios, such as the granulocyte-to-lymphocyte ratio (GLR), the lymphocyte-to-monocyte ratio (LMR) and the platelet-to-lymphocyte ratio (PLR) [11]. Many studies have shown that such markers as the neutrophil-to-lymphocyte ratio (NLR), PLR and LMR may predict systemic inflammation and may be useful in the diagnosis of many diseases [12,13]. The markers are mainly used in clinical studies on cancer [14] and liver disease [15] and are less often used in sports diagnostics [16]. Heart failure (HF) and cardiovascular disease, the risk of which increases during perimenopause, are not considered isolated conditions, but rather complex systemic disorders. Whatever the pathogenesis of HF, the main cause of its progression is cardiac remodelling. Heart-infiltrating cells, including granulocytes, monocytes, macrophages, dendritic cells, mast cells and lymphocytes T and B, cause the release of cytokines that alter the inflammatory response and the remodelling of the myocardial extracellular matrix [17].

Systemic inflammation leads to changes in the number of neutrophils, lymphocytes, monocytes and platelets [18]. Many studies have shown that NLR, PLR and LMR may predict systemic inflammation, being useful in many diseases. Thus, LMR may be a useful marker of disease development and mortality risk [12,19]. In addition to biochemical markers, such anthropometric parameters as the waist-to-hip ratio (WHR), visceral fat area (VFA) and the visceral adiposity index (VAI) are often used to assess the risk of cardiovascular disease [20]. VAI has been shown to have good predictive power for the visceral adiposity-related risk of type 2 diabetes and hypertension. It should be seen as an indicator of changes in adipose function associated with the development of insulin resistance. Therefore, VAI can be a good predictor of the risk of cardio- and cerebrovascular events [21,22].

Findings from studies undertaken in recent years indicate that the coefficient of variation red blood cell distribution width (RDW-CV), too, has a high prognostic value in assessing the risk of cardiovascular disease [23]. The greater the variability in red blood cell size, the higher the RDW-CV values. Research has shown an unfavourable prognostic value of RDW-CV alone, as well as in combination with other parameters, in heart failure [24] coronary heart disease [23,25] and myocardial infarction [26].

It can be concluded that studies undertaken over the last 20 years have provided solid evidence that the risk of cardiovascular disease increases in perimenopausal and postmenopausal women, irrespective of age-related changes. Therefore, it is necessary to develop effective preventive strategies to reduce cardiometabolic risk factors and thus, reduce the risk of cardiovascular events in perimenopausal and older women, previously lowering body fat mass, improvement of lipid profile, reduction of blood pressure or systemic inflammation.

Engaging in the amount of moderate physical activity recommended by the World Health Organization (WHO) helps reduce the risk of death and adverse health events both in healthy individuals and those with cardiovascular disease [27,28]. Regular physical activity (both endurance and combined endurance and resistance training) reduces body weight, improves cardiopulmonary fitness and reduces inflammation. Aqua fitness training involves moderate-intensity exercise (according to the European Society of Cardiology: 3.0–5.9 METs) performed in water whose properties such as buoyancy, increased hydrostatic pressure and temperature enhancing thermoregulatory processes, are beneficial to health [29,30]. This type of physical activity is preferred by many people with poorer motor skills [31,32]. Aquatic exercise improves glucose metabolism and lipid profile and reduces body weight, blood pressure and pain reduction [33,34,35].

The aim of the present study was to assess the effect of a 6-month aqua aerobics programme on cardiometabolic (anthropometric and biochemical) parameters in perimenopausal women.

## 2. Materials and Methods

### 2.1. Participants

Fifty women who responded to a local advertisement and were deemed medically fit to participate in the programme were recruited for the study. The study included perimenopausal women who were in sufficiently good health to participate in the training programme. Women with neuromuscular disorders affecting their ability to move independently, morbid obesity, chronic inflammatory disease, active or recent cancer or unstable coronary heart disease and women after myocardial infarction (<12 months) or stroke (<6 months) were excluded from the study. The study also excluded women with mental disorders, severe respiratory failure or skin diseases preventing participation in aquatic exercise. Women receiving steroid therapy or addicted to medication, drugs or alcohol were, too, excluded from the study.

Before the study, participants were advised not to change their eating habits and not to participate in any other sporting activities during the project. All participants reported that they did not take part in competitive sports and rated their level of physical activity as moderate. Forty women took part in the first testing session and were then randomly assigned to the control (*n* = 20) and study (*n* = 20) groups. A person not associated with the research project made a random allocation to the groups. She used a computer list in Excel software. Ultimately, 16 women in the control group and 14 women in the study group who completed the 6-month training programme took part in the second testing session. Women in the study group were required to attend at least 80% of the planned training sessions. The study was conducted in accordance with the Declaration of Helsinki and was approved by the Ethics Committee of the IRB at the Poznan University of Medical Sciences (decision No. 882/11). Participants could withdraw from the study at any time without giving a reason.

### 2.2. Anthropometric Measurements

Basic tests included anthropometric measurements, blood pressure measurement and blood biochemical testing and were performed following standard procedures by authorised persons. The tests were performed twice, i.e., at the beginning (first testing date) and at the end (second testing date) of the project. Body weight, BMI and body composition (FM, MM, VFA) were measured using the bioimpedance method with the use of the Tanita MC-780 MA analyser (Tanita Corp., Tokyo, Japan). All measurements were performed in accordance with the manufacturer’s guidelines related to the reliability of the method. Height was measured using the WPT 60/150 OW medical scales with a height measuring rod (Radwag^®^, Radom, Poland). Waist and hip circumferences were measured in accordance with relevant measurement guidelines using a tape measure (accuracy: +/− 1 cm). Based on the measurements, the waist-to-hip ratio (WHR) was calculated.

### 2.3. Preparation of Blood Samples for Analysis

Complete blood count and lipid profile analyses were performed in a certified diagnostic laboratory in Kalisz. Blood samples of 10 mL were taken from the ulnar vein (fasting blood test) in the morning using an S-Monovette syringe (Sarstedt, Nümbrecht, Germany). The following haematological and biochemical parameters were determined: haemoglobin concentration (Hb), haematocrit value (HCT), white blood cell count (WBC), total red blood cells count (RBC), red blood cell distribution width–coefficient of variation (RDW-CV), total cholesterol (T-C), high-density lipoprotein cholesterol (HDL-C), low-density lipoprotein cholesterol (LDL-C), triglyceride levels (TG).

Based on the anthropometric and biochemical measurements, the platelet-to-lymphocyte ratio (PLR), the lymphocyte-to-monocyte ratio (LMR) and the visceral adiposity index (VAI) were calculated. VAI was calculated in accordance with the following formula: VAI = (WC(cm)/(36.58 + (BMI ∗ 1.89) ∗ (TG/0.81) ∗ (1.52/HDL). It is a sex-specific empirical–mathematical method. According to a publication by Amato et al., the cut-off points of VAI for women aged 42–52 are as follows: ≤1.92—no adipose tissue dysfunction (ATD), 1.93–2.15—mild ATD, 2.17–2.77—moderate ATD, >2.77—severe ATD [21].

### 2.4. Training Programme

The project lasted 6 months and included 24 once-weekly training sessions. During the training sessions, participants wore flotation belts, which enabled them to safely perform aquatic exercises in deep water without the feet being in contact with the bottom of the pool. The exercises were carried out to music and were led by a qualified instructor, who was standing at the edge of the pool, providing a detailed demonstration of each exercise. Different types of resistance equipment were used during the training sessions (long pool noodle, short pool noodle, BEtomic, aqua disc, gloves, cuffs, happy flower, big wave bells, punches).

Each training session lasted 45 min and consisted of three parts, during which specific exercises were performed:

1. warm-up (walking in place, arm exercises in different planes) warm-up/cardio (running in place, running in multiple directions, arm exercises with different hand positions, movement exercises in different directions);
2. main part (aerobic/strengthening) (arm exercises in multiple directions and with different ranges of movement (pushing/scooping), leg exercises (single- and double-leg raises, jumps, jumping jack, scissors, grounded, elevated), coordination exercises);
3. cool-down (exercises in a front-lying position, exercises in a back-lying position, position change exercises, stretching and relaxing exercises).

The training programme was developed based on methodological guidelines for conducting aqua aerobics classes and was appropriate to the ability of participants. The determinant of training capacity is the resting heart rate and age of the participants. During training, heart rate values oscillated between 40 and 70% of the maximum heart rate value. We calculated the maximum heart rate according to the formula MHR 220–age (in years), keeping in mind the hydrostatic pressure of water, by the influence of which the heart rate of the participant in the aquatic environment is 10 to 20 beats slower than on land. The volume of classes ensures that the expected training effects are achieved. [36,37].

### 2.5. Statistical Analysis

Data were analysed statistically using Dell Statistica software (version 13, software.dell.com, Dell Inc., Round Rock, TX, USA). The normality of the distribution of variables was tested using the Shapiro–Wilk test. The Mann–Whitney U-test was used for inter-group comparisons, whereas the Wilcoxon test was used for intra-group comparisons. Relationships between variables were analysed using Spearman’s rank analysis. For statistically significant changes, effect sizes (ES) were calculated and assessed against Cohen’s criteria [38]. Data are presented as means and standard deviations (SD). Statistical significance was set at *p* ≤ 0.05. Using TG concentration data from the manuscript by Volakis et al. [39], after calculating the power analysis of the Mann–Whitney test (taking power as 1—beta error probability: 80%, effect size: 0.98, and error taken as alpha: 0.05 (two-sided)), 15 participants were identified for analysis.

## 3. Results

A total of 30 women (16 women in the control group and 14 women in the study group) fulfilled the criteria adopted and completed the 6-month study. Table 1 shows the values of anthropometric parameters of cardiovascular risk and blood pressure values.

The group studied was homogenous in terms of the anthropometric and biochemical characteristics analysed. On the first testing date, no significant differences were observed in the variables between the study group and controls. The women participating in the study were slightly overweight; the mean BMI for the study group was 27.19 kg/m^2^, whereas the mean BMI for the control group was 25.59 kg/m^2.^ Both groups had normal visceral fat area (VFA) and blood pressure levels. Moreover, both groups had similar, normal VAI values. Haematological parameters and lipid profiles of the women participating in the study on both testing dates are shown in Table 2. The groups were also homogenous in terms of the aforementioned parameters on the first testing date. Hb concentration, TG and HDL-C levels and RBC counts, WBC counts, PLT counts and RDW-CV values were within the normal range. However, both groups had elevated T-C levels on the first testing date.

The 6-month aqua aerobics programme resulted in a significant decrease in the waist-to-hip ratio (*p* < 0.05; ES: 2.143), diastolic blood pressure (DBP) (*p* < 0.05; ES: 1.005) and platelet-to-lymphocyte ratio (*p* < 0.05; ES: 0.460) and an increase in HGB concentration (*p* < 0.05; ES: 0.643). In the control group, there was a significant reduction in body weight (*p* < 0.05; ES: 0.175), BMI (*p* < 0.05; ES: 0.210) and VFA (*p* < 0.05; ES: 0.304) and an increase in RDW-CV values (*p* < 0.05; ES: 0.444). A statistical analysis of differences between the groups in the changes in the parameters analysed between the first and second testing dates showed a significant difference between the groups in the change in the waist-to-hip ratio (∆ WHR) (*p* < 0.05; ES: 2.705).

In the first period of the study (at the beginning of the training programme), correlations were observed between TG levels and WBC counts (r = 0.5390, *p* < 0.05), between TG levels and Hb concentration (r = 0.5265, *p* < 0.05), between WBC counts and DBP (r = 0.6098, *p* < 0.05) and between age and VFA (r = 0.6367, *p* < 0.05). An analysis of relationships between changes in the parameters analysed between the first and second testing dates for the whole group studied showed a relationship between the change in TG levels (∆ TG) and the change in body weight (∆ body weight) (r = 0.4386, *p* < 0.05), between the change in TG levels (∆ TG) and the change in VAI (∆ VAI) (r = 0.8973, *p* < 0.05), between the change in RDW-CV (∆ RDW-CV) and the change in systolic blood pressure (∆ SBP) (r = 0.3912, *p* < 0.05), between the change in the lymphocyte-to-monocyte ratio (∆ LMR) and the change in BMI (∆ BMI) (r = −0.4053, *p* < 0.05) and between the change in the waist-to-hip ratio (∆ WHR) and the change in body weight (∆ body weight) (r = −0.4135, *p* < 0.05).

## 4. Discussion

The aim of this randomised controlled study was to analyse the effect of a 6-month aqua aerobics programme on cardiometabolic (anthropometric and biochemical) parameters in perimenopausal women. We analysed the anthropometric parameters of cardiovascular risk such as VFA, WHR and VAI, SBP and DBP levels as well as blood biochemical parameters, including, among others, lipid profile, RDW-CV and inflammation markers such as PLR and LMR, which are considered by some as risk factors for heart disease. The women who took part in the 6-month training programme (45 min of exercise per week) had a lower WHR (*p* < 0.05), lower DBP (*p* < 0.05), lower PLR (*p* < 0.05), lower LDL-C levels (*p* < 0.05) and higher Hb levels (*p* < 0.05) compared with controls on the second testing date. The changes in these parameters can be considered to be beneficial as regards the risk of morbidity, and the form of training itself, with its low frequency and intensity, can be considered as contributing to the maintenance of the physical well-being of perimenopausal women.

Lack of physical activity or insufficient intensity or frequency of physical activity is associated with lower overall quality of life—and not only in older people. The hormonal and metabolic changes resulting from the ageing of ovaries and reduction in oestrogen are well described in the literature, as is the increase in the risk of cardiovascular disease in this group of people [40]. Exercise is a non-pharmacological way of controlling proatherosclerotic lipid levels, inflammatory markers and other cardiovascular risk factors [41,42]. The type of exercise and its intensity should be tailored to each individual’s preferences and health. Moreover, the choice of exercise should take into account the individual’s health limitations that prevent him or her from performing certain types of exercise. In obese or overweight individuals, traditional forms of training, such as walking training or exercise on a cycle ergometer, can cause a number of musculoskeletal complications, which may discourage them from continuing to participate in longer-term training programmes [43]. Aquatic training has been found to stimulate peripheral vasodilatation and blood flow redistribution. Increased atrial pressure stimulates low-pressure cardiopulmonary receptors. Moreover, it inhibits sympathetic nervous activity. The physiological changes associated with immersion in water may have positive health effects as regards circulatory fitness [43,44,45]. This was confirmed by the findings from the present study, which showed that a 6-month aqua aerobics programme with once-weekly training sessions resulted in a significant decrease in DBP (*p* < 0.05). Aquatic training does not cause undue stress on joints and immersion in an upright position stimulates the secretion of atrial natriuretic peptides, which contribute to increased lipid oxidative capacity. During the exercise, the level of lipid mobilization and the speed of lipid oxidation remain unchanged or even increase despite the reduced activation of the sympatho-adrenal system. The processes and relationships described above result in improvement in lipid profile [46,47]. Our study showed that women in the study group who participated in a 6-month supervised aquatic training programme had lower LDL-C levels on the second testing date compared with controls (*p* < 0.05). This was probably due to the physical activity-dependent change in intravascular enzyme activities described in a publication by Weise et al. [48]. Increased lipoprotein lipase activity (LPLA) and lecithin-cholesterol acyltransferase (LCAT) activity (LCATA) and reduced hepatic TG lipase activity (HLA) and cholesterol ester transfer protein (CETP) concentration may result in a decrease in TG levels and an increase in HDL-C levels.

There is extensive debate about the effectiveness of anthropometric measurements in identifying the risk of cardiovascular disease. Undoubtedly, high body weight, especially high visceral fat mass, is a strong predictor of future circulatory disease. From a clinical point of view, parameters based on many anthropometric and biochemical measures, such as VAI, should be highly effective in predicting the risk of cardiovascular disease. In the present study, we found no significant change in VAI in the women participating in the aquatic training programme. However, the training programme resulted in a statistically significant decrease in WHR, DBP and LDL-C levels. In 2012, Mohammadreza et al. published the findings from their 9-year observational study analysing the effectiveness of VAI in predicting cardiovascular disease compared with simple anthropometric measures of obesity, i.e., BMI, waist-to-height ratio and waist-to-hip ratio [49]. They found that VAI is independently associated with increased risk of incident cardiovascular disease in women and that the level of this risk is not significantly higher than the level of risk due to BMI, waist-to-height ratio or waist-to-hip ratio. However, the authors found no association between VAI and any significant increased risk of incident cardiovascular disease among men. Thus, the use of this parameter alone is ineffective and may provide misleading or incomplete information.

A number of studies have found that variation in RBC size has a significant impact on the severity of cardiovascular disease caused by changes in blood flow through vessels. Changes in blood flow lead to interactions between morphotic elements and the vascular endothelium [50], which results in the development of inflammation, which leads to the development of atherosclerosis. The literature on the effect of certain forms of exercise on RDW-CV is limited, making it difficult to correctly interpret changes in RDW-CV values [51]. In the present study, a 6-month aqua aerobics programme did not result in a significant change in RDW-CV, even though participants in the study group had lower LDL-C levels on the second testing date compared with the control group. This was most probably due to the fact that the training was of too low intensity and frequency.

The blood biomarkers associated with inflammation analysed in the present study play an important role in the development of many diseases and can be used to assess the risk of disease development or progression. In the present study, we found a decrease in such parameters as NLR and PLR in the group studied (*p* < 0.05). Undoubtedly, the fact that the women studied already had low inflammation levels on the first testing date may also be due to their overall good health, normal body weight and normal blood glucose levels, and the changes observed in the women who participated in the training programme are undoubtedly favourable. The NLR and PLR values reported in the literature in, for instance, people with diabetes, are higher, which is associated with higher glucose levels and high insulin resistance [52,53,54].

## 5. Conclusions

The type of physical activity described in the present study is a great way for perimenopausal women to take care of their overall well-being. The favourable changes observed on the second testing date in the women who participated in the training programme may result in a reduction in the risk of cardiovascular disease. An important aspect is the low, but effective, frequency of exercise. Short training programmes very often do not bring spectacular results, whereas those lasting several months can often be discouraging. It should be noted that the women who participated in the training programme as part of the present study were perimenopausal and many of them were employed. Thus, it is difficult for them to engage in regular exercise (2–3 times per week) for a longer period of time. Therefore, the demonstrated effectiveness of the training programme and the reduction in selected cardiometabolic parameters are important from the point of view of the protection of women’s health. The question arises as to whether this form of physical activity would also be effective in older people, including men, and whether it could compensate for the loss of physiological reserve associated with ageing.

## Figures and Tables

**Table 1:** Values of anthropometric parameters, blood pressure values and VAI for the study and control groups on the first and second testing dates.

	Control Group (*n* = 16)			Study Group (*n* = 14)		
	Baseline	6 Months	*p*-Value	Baseline	6 Months	*p*-Value
Age (year)	47.00 (7.00)			48.43 (6.72)		
Body weight (kg)	69.44 (12.36)	67.34 (11.67)	0.0174 (ES: 0.175)	70.87 (10.49)	70.85 (11.11)	0.6377
Body height (cm)	164.44 (6.79)			161.36 (6.26)		
BMI (kg/m^2^)	25.59 (3.65)	24.83 (3.59)	0.0106 (ES: 0.210)	27.19 (3.24)	27.17 (3.57)	0.7007
FM %	32.80 (7.21)	30.60 (5.64)	0.1034	34.66 (4.65)	33.51 (5.68)	0.1405
MM %	43.64 (4.46)	43.91 (5.43)	0.7332	43.52 (4.17)	48.66 (4.16)	0.3152
VFA	5.87 (2.22)	5.25 (1.84)	0.0277 (ES: 0.304)	6.71 (1.98)	6.57 (1.99)	0.5286
WHR	0.90 (0.07)	0.90 (0.07)	0.9096	0.94 (0.07)	0.79 (0.07)	0.0009 (ES: 0.143)
VAI	3.25 (1.92)	3.57 (2.64)	0.3010	3.96 (4.56)	3.66 (3.67)	0.5098
SBP (mmHg)	116.25 (14.32)	112.50 (14.26)	0.2132	123.21 (9.12)	115.00 (15.19)	0.0917
DBP (mmHg)	77.50 (10.65)	75.00 (10.80)	0.3669	86.79 (6.96)	78.93 (8.59)	0.0185 (ES: 1.005)

Data are presented as mean (SD), BMI—body mass index, FM—fat mass, MM—muscle mass, VIS—visceral fat area, WHR—waist–hip ratio, VAI—visceral adiposity index, SBP—systolic blood pressure, DBP—diastolic blood pressure.

**Table 2:** Haematological parameters and lipid profile of women in the control and study groups on the first and second testing dates.

	Control Group (*n* = 16)			Study Group (*n* = 14)		
	Baseline	6 Months	*p*-Value	Baseline	6 Months	*p*-Value
WBC (10^9^/L)	6.37 (1.61)	6.32 (1.47)	0.7764	6.74 (2.16)	7.31 (2.11)	0.0868
RBC (10^12^/L)	4.36 (0.28)	4.30 (0.28)	0.2805	4.46 (0.25)	4.52 (0.25)	0.0901
HGB (mmol/L)	13.05 (0.79)	13.09 (0.89)	0.3152	13.22 (0.96)	13.88 (1.09)	0.0013 (ES: 0.643)
PLT (10^9^/L)	227.56 (51.13)	238.12 (42.34)	0.0703	245.93 (39.29)	244.79 (30.09)	0.8339
RDW-CV (%)	13.79 (0.46)	14.03 (0.61)	0.0202 (ES: 0.444)	13.75 (0.95)	14.06 (0.29)	0.1961
PRL	104.48 (29.38)	103.84 (23.58)	0.8361	98.66 (20.27)	89.58 (19.16)	0.0219 (ES: 0.460)
LMR	8.43 (2.64)	7.74 (1.64)	0.7959	7.94 (3.16)	7.89 (1.59)	0.5936
TC (mg/dL)	216.87 (22.16)	212.62 (24.80)	0.5895	217.93 (35.97)	202.92 (32.32)	0.0868
HDL-C (mg/dL)	72.12 (14.29)	71.00 (15.81)	0.6247	70.43 (16.55)	69.07 (17.04)	0.5525
LDL-C (mg/dL)	123.62 (28.61)	120.62 (28.83)	0.6603	125.14 (28.71)	114.00 (27.04)	0.0277 (ES: 0.399)
TG (mg/dL)	105.69 (50.36)	105.31 (51.79)	0.9176	113.07 (91.92)	117.79 (82.34)	0.6605

Data are presented as mean (SD), WBC—white blood cell count, RBC—red blood cell count, HGB—haemoglobin concentration, PLT-—platelets, RDW-CV—coefficient of variation red blood cell distribution width, PRL—platelet/lymphocyte ratio, LMR—lymphocyte/monocyte ratio, TC—total cholesterol, HDL-C—high density lipoprotein cholesterol, LDL-C—low density lipoprotein cholesterol, TG—triglycerides.

## Data Availability

Not applicable.
